# The Evaluation and Optimization Methods of Villages in China: In the Background of a Rural Revitalization Strategy

**DOI:** 10.1155/2022/7314446

**Published:** 2022-08-24

**Authors:** Ruyi Zhang, Chenxi Ma, Degang Wu, Yinliang Wu, Ke Wang

**Affiliations:** ^1^Zhejiang Urban Construction Planning and Design Institute, Hangzhou, China; ^2^China Academy of Urban Planning & Design, Beijing, China; ^3^Faculty of Civil Engineering and Architecture, Zhejiang University of Science & Technology, Hangzhou, China; ^4^Faculty of Forestry and Biotechnology, Zhejiang A&F University, Hangzhou, China; ^5^Zhejiang University, Hangzhou, China

## Abstract

Over the past few decades of rapid development, China has always attached great importance to the redevelopment of rural areas. Since the 19th National Congress of the Communist Party of China, the rural revitalization strategy proposed at the meeting has always received extensive attention. In 2019, the Chinese central government released a document on its rural revitalization strategy. In the document, Guizhou Province was listed as a key area to receive focus on pursuing rural revitalization. Meanwhile, the Guizhou government also formulated The *Implementation Opinions on the People's Government of Guizhou Province's Rural Revitalization Strategy*. The aim of this paper is to try to establish a model according to the rural revitalization strategy proposed by the Guizhou government and extracts the main influencing factors from its analysis. Through the analysis of the data collected during the process of the rural revitalization, the paper undertakes a deep analysis of the evaluation and studies an optimized approach to the revitalization to provide a referenced review for other villages.

## 1. Introduction

Rural decline, as a global issue, is inevitably accompanied by increasing global levels of urban development [1]. Even if China's urbanization rate will reach 60% or higher in the future, nearly 500 to 600 million people still live in the countryside, providing agricultural products and services to 1.4 to 1.5 billion people [2]. Most of China's rural areas are still in a state of decline, differing from those in Western countries. The situation regarding the economy and resources regarding transportation, education, and medical care is limited, especially in Qiandongnan Miao and Dong Autonomous Prefecture, which is the focus of the research. Most of Guizhou Province lies in mountainous areas, so there are as many as 409 traditional villages in the prefecture, accounting for 56.41% of the whole area. The area has dense and numerous traditional villages, with distinctive ethnic cultural characteristics. It is an iconic example of villages in Guizhou Province [3]. It is also home to 33 ethnic groups such as Miao, Dong, and Han, so many villages retain the primitive spatial layout and architectural form [4]. The protection and redevelopment of these traditional villages has not received enough attention in the past few decades, so the task of rural revitalization is pressing. In the 19th National Congress of the Communist Party of China, *The Opinions of the Central Committee of the Communist Party of China and the State Council on Implementing the Strategy of Rural Revitalization* has been repeatedly emphasized as the outline for rural construction, which has been “No. 1 Central Document” since the central government focused on issues concerning agriculture, countryside, and farmers in 2018. *The Strategic Plan for Rural Revitalization (2018–2022)* was reviewed and released by the central government and the Political Bureau of the CPC Central Committee [5]. The document indicates that the goals are to build areas with thriving industries, sound water conservancy facilities, put in place sound systems and mechanisms for promoting integrated urban-rural development, and to improve basic work in rural areas, which can be summarized as *the 20-character policy* for rural revitalization [5]. To implement the strategic deployment of the 19th Party Congress, Guizhou Province implemented an action plan for a comfortable life in 2018 in line with these strategies and achieved remarkable results: the per capita disposable income of permanent residents increased by 6% to 10%. Sixteen poverty-stricken counties and 2,500 poverty-stricken villages were lifted out of poverty. The number of rural people living below the poverty line was reduced by more than 1.2 million. The water supply coverage has reached more than 78%. Power supply reliability has reached 99.7%. As a result, the province has a more than 700,000 strong rural labor force to achieve the transfer of employment, including 250,000 migrant workers returning to their hometowns for business startups and employment. On July 18 of the same year, the Central Government of China compiled *The Implementation Opinions of the Guizhou Provincial Committee of the Communist Party of China and the People's Government of Guizhou Province on the Rural Revitalization Strategy*, which is mainly focused around *the 20-character policy* “thriving industries, pleasant living environments, social civility and etiquette, effective governance, prosperity” for rural revitalization.

Based on the guiding theory put forward in this document and the data obtained by fieldwork, this article collects data through in-depth field investigations in various villages, the paper established an evaluation system for rural revitalization planning in Qiandongnan Miao and Dong Autonomous Prefecture through interviews with farmers and village committees and data collection from deep field studies. Through the system, we found out how a series of policies for rural revitalization has affected the development of traditional villages in the area recently, and we sum up what can reflect the core values and what cannot from the data.

In the next section, a brief literature review is carried out based on the realities of how villages develop in Guizhou Province [6] (Guizhou Provincial People's Government's Guiding Opinions on Strengthening the Protection and Development of Traditional Villages, 2015-5-6. https://www.gzgov.gov.cn/gzxw/739177.shtml), the development history, and basic laws of most traditional villages in China. The fourth section mainly introduces the research method. The fifth section summarizes the optimal approaches of village planning through the data obtained from the research. The last section summarizes the findings and provides suggestions.

## 2. Materials and Methods

### 2.1. Literature Review

#### 2.1.1. Basic Laws of Village Development

In the past few decades, the rapid development of urbanization in China and the rapid expansion of cities have encouraged many young people to move from rural areas to big cities for more opportunities [7], thus resulting in many traditional and culturally historic villages becoming victims of “Village-hollowing,” also known as “Dying Villages” in Europe [8]. In the past 20 to 30 years, apart from agricultural development, rural planning has changed from agricultural-based to mutifunctional and environmental planning [9, 10]. Therefore, many traditional Chinese villages have developed a combination of primary, secondary, and tertiary industries according to their own characteristics. For example, some villages have developed a new form of tourism based on village tours, which has emerged in the past decade. It has played an important role in increasing farmers' income and human capital, promoting local employment, improving rural economic structure, protecting rural environment, and inheriting rural traditions [11]. Undoubtedly, villages often need tourism industries to drive their local development [12]. While the documents about countryside tourism suggest that the new form drives local employment, such employment is often limited to the sale of handicrafts, cultural performances, hospitality and accommodation, and low-consumption services. In the case study of Liuzhuanghu Village in Beijing, Li et al. found that the village collective is very important to the full participation of village enterprises in the formulation of village planning in the early stage of village planning [13]. At the same time, government departments should promote the coconstruction, sharing, and intensive use of industrial land [14]. Since 2006, most of rural areas in China have been influenced by “Building a New Socialist Countryside” campaign, which was aimed to reduce the income gap between urban and rural residents [15]. As a result, the policy directly improved rural live standards [16].

#### 2.1.2. Rural Revitalization Promoting the Development of Traditional Villages

In China, the concept of traditional villages originated at the end of 2012. The Ministry of Housing and Urban-Rural Development, the Ministry of Culture, and the Ministry of Finance jointly issued guidance on Strengthening the Protection and Development of Traditional Villages, calling for the strengthening of the protection and development of traditional villages, maintaining rural characteristics, making the countryside more attractive, and injecting new economic vitality into rural areas. Since then, the protection of traditional villages has been valued nationally. By the end of 2016, a total of more than 4,000 villages were listed as ethnically traditional villages. Some of these villages are listed as World Cultural Heritage Sites and receive tourists from all over the world, such as the ancient town of Xidi, Hongcun town, Kaiping Watchtower and Villages, and so on [17]. These villages have great cultural, historical, artistic, and architectural value. In ancient times, there was no huge difference between urban and rural areas. Compared with rural areas, cities did not have noticeable advantages. The lifestyle, culture, and taste did not show too much superiority and individuality. Indeed, bureaucrats and wordsmiths regarded cities as places of hustle and bustle or cages shielding people from different political and life perspectives. They even had the idea of loving the countryside and hating cities, forming a far-reaching reclusive view [18]; in addition, their impact on the landscape whether positive or negative is often unintentional [19]. However, due to the poor development of traditional villages and the lacking of rural infrastructure, more and more young people moved to big cities to seek more development opportunities and better education, medical care, etc. Many of them gave up their houses and farmland. As a result, many villages with hundreds of years of cultural history became hollow villages inhabited mostly by the elderly and just a few children. But in recent years, more and more scholars have become more and more interested in these traditional villages. The Chinese government has also formulated a series of related policies to protect these villages [20]. On August 21, 2014, the State Council issued the “Several Opinions of the State Council on Promoting the Reform and Development of Tourism,” which called for strengthening targeted poverty alleviation, solidly promoting the project of enriching people through rural tourism and promoting poverty-stricken areas to get rid of poverty and create wealth. From the policy, it is implied that the development of rural tourism can promote economic growth. In 2012 alone, the labor force employed in rural tourism increased by more than 13 million, and the growth rate of rural labor employment reached 86.7%. Rural tourism not only promotes the development of tourism and agriculture but also provides various employment channels and opportunities for the residents [21]. Due to industrial integration, it provides a new way of upgrading the agricultural industry and boosts farmer and agricultural workers' incomes. It increases the industrial range of agriculture and expands the industrial system of agriculture. The diversified development of village industries is in line with the demand of practice and the direction that is taking, especially the integration of the primary industry and the tertiary industry, which is conducive to the full play to functional advantages of agricultural products and services, such as the appearance of rural experiences, and creative agriculture.

#### 2.1.3. Rural Revitalization Guided by Industrial Development Planning

The decline of rural areas around the world is a common phenomenon caused by industrial civilization and the development of modern society. However, in the postmodern world, rural areas are not only places for agricultural production but also ones for leisure, tourism, specialty food production, and consumption [22]. Now, traditional ways of lifestyle, such as crop cultivation, fishing, or forestry, cannot achieve the goal of helping to promote rural development and help rural residents get out of poverty. As early as the 1980s, the Sustainable Livelihoods Approach (SLA) was proposed, a comprehensive theory of rural sustainable development [23]. In China, agricultural-related rural poverty issues are fundamental issues related to people's livelihoods. Therefore, it is necessary to integrate rural life into the development of rural industries. In other words, rural ecological natural resources should be fully protected and utilized, and rural historical and cultural resources should be inherited and taken advantage of. Taking rural ecological “tourism and vacation +” as the leader, primary and secondary industries such as ecological agriculture, creative culture, and agricultural product processing should be integrated to form new activities and consumption scenarios such as “tourism vacation + ecological agriculture sightseeing,” creative cultural experience, and agronomic processing experience. We should insist on ecological sustainability and the integrated development of the primary, secondary, and tertiary industries by the drive of tourism [24]. The development process of China's rural industries can be summarized into two stages. The first stage is rural industrialization—the primary production mode of small-scale farming transformed into a mixed mode of industry and agriculture with the development of rural enterprises thanks to the help of villages collectively. The second stage is industrial transformation: agriculture and tourism develop decaying industries together. The village industry development plan is to make quantitative and qualitative arrangements for villages' tertiary industries according to the current situation with the development of agriculture, secondary and tertiary industries, the resource endowment of production factors, and market development needs. Village industrial development planning should see industries as the main driver for rural development to arrange industrial development and spatial configurations, not only from land use [25].

Numerous case studies have implied that it is vital to rely on the unique resources of villages to develop secondary and tertiary industries for solving rural industrial and economic problems. For example, through 8 years of research and practice on rural revitalization in the village of Yuanjiacun, Shaanxi Province, Yuanjiacun has found that being a rural tourism hotspot has helped develop its tofu industry, and its brand is now well-known also thanks to its unique development and support from government policies, thus gradually developing it into a relatively wealthy area. Even for some villages with no natural resources and historically significant culture at all, it is still possible to change the fate of the entire village by creating unique industrial clusters as an effective way of rural revitalization [26]. For example, Dafen Village is an example of “best practice” governance because it has transformed itself from a backward village into an artistic hub [27].

#### 2.1.4. Research Ideas and Methods

The focus of this study is to build an element evaluation system, selecting representative rural areas in southeastern Guizhou to measure and analyze the comprehensive development level of high-quality rural development, to provide an evaluation reference for Guizhou, and to provide a relative comparison for the whole country. In order to make the evaluation system meet the needs of theory and practice, this study fully draws on the selection and coefficients of other scholars in terms of relevant elements. This study measures the representative rural development of southeastern Guizhou on five levels: thriving industries, social civility and etiquette, prosperity, effective governance, and pleasant living environments. The above five levels have corresponding element measurement systems. The data used in this study is expected to systematically and comprehensively reflect the key factors of realizing rural revitalization, so the factors are strictly selected. In order to determine the proportion of each factor, the entropy weight method and factor analysis method is used to determine the result. Finally, total scores for villages are achieved through the TOPSIS method to assess the development. Based on the scores, a comparison is made to draw an objective, fair, and valuable evaluation.

## 3. Results and Discussions

### 3.1. Models

This study uses the factor analysis to extract the main influencing factors, which can avoid the influence of multicollinearity to a certain extent so as to determine the main factors affecting the high-quality development of rural areas. The variance contribution of the main factors and the coefficient component score are changed to determine the weight of each element by a difference coefficient of the entropy method, so that the evaluation results are objective, fair, and valuable [28]. Finally, the comprehensive scores of representative villages are calculated by the TOPSIS method, which avoids the simple average summation method. Using these steps to get the comprehensive scores for assessing the rural development make the results more logical and meet the demands of the present situation more closely. The specific method of calculation is as follows:

**Step 1 .**

Build positive definite matrices with multiple elements. Based on existing references and the present situation, suppose there are *m* proposals to evaluate and analyze and there are *n* elements. So, a data matrix *X*=(*x*_*ij*_)_*m*×*n*_ is formed.

For positive elements:

(1) $$\begin{array}{c} X_{ij}^{\prime }=\frac{X_{ij}-\min\left(X_{1j},X_{2j},...,X_{ni}\right)}{\max\left(X_{1j},X_{2j},...,X_{ni}\right)-\min\left(X_{1j},X_{2j},...,X_{nj}\right)},1\leq i\leq m,\;1\leq j\leq n;\frac{n!}{r!\left(n-r\right)!}. \end{array}$$

For negative elements:

(2) $$\begin{array}{c} X_{ij}^{\prime }=\frac{\min\left(X_{1j},X_{2j},...,X_{ni}\right)-X_{ij}}{\max\left(X_{1j},X_{2j},...,X_{ni}\right)-\min\left(X_{1j},X_{2j},...,X_{nj}\right)},1\leq i\leq m,1\leq j\leq n. \end{array}$$

**Step 2 .**

Determine the entropy value of every element and the component score coefficient. The entropy value of every element is calculated by standardized data and then the component score coefficient by the entropy value.

The entropy value *e*_*j*_=−∑_*i*=1_^*m*^*b*_*ij*_ln  *b*_*ij*_/ln  *m*, where *b*_*ij*_ is the weight of *j* on *i*, *b*_*ij*_=*x*_*ij*_′/∑_*i*−1_^*m*^*x*_*ij*_′. After getting the entropy value *e*_*j*_, the component score coefficient *g*_*j*_, *g*_*j*_=1 − *e*_*j*_, is calculated by the entropy value.

**Step 3 .**

Figure out the weight coefficient by factor analysis. The contribution of variance *p*_*k*_ and component score coefficient *β*_*i*_ is calculated by factor analysis. Suppose there are *y* common factors, and then the weight of every element is *w*_*j*_=*p*_*k*_*β*_*j*_*g*_*j*_/∑_*j*=1_^*n*^*p*_*k*_*β*_*j*_*g*_*j*_, 1 ≤ *j*, *k* ≤ *n*.

**Step 4 .**

Build the weighted matrix with elements. The weight matrix [*w*_1_, ..., *w*_*n*_]^*T*^ can be obtained from step 3. Normalized matrix *X*=(*x*_*ij*_)_*m*×*n*_ is based on the weight matrix and step 1 so as to achieve *Q*=(*q*_*ij*_)_*m*×*n*_, and *q*_*ij*_=*x*_*ij*_*w*_*j*_.

**Step 5 .**

Calculate the total scores of representative villages. The element weighting matrix *Q*=(*q*_*ij*_)_*m*×*n*_ obtained in step 4 can calculate the optimal solution and the worst solution of each element.

The optimal solution is *A*^+^={max*A*_*ij*_*|i*=1,…, *n*}={*a*_*i*1_^+^, *a*_*i*2_^+^,…, *a*_*in*_^+^}; and the worst is *A*^−^={max*A*_*ij*_*|i*=1,…, *n*}={*a*_*i*1_^−^, *a*_*i*2_^−^,…, *a*_*in*_^−^}.

Then, calculate the gap between the totals and the optimized solution and the gap between the totals and the worst solution.

Gap between the totals and the optimized solution is $D^{+}=\sqrt{\sum_{j=1}^{n}{\left(q_{ij}-a_{ij}^{+}\right)}^{2}}$.

Gap between the totals and the worst solution is $D^{-}=\sqrt{\sum_{j=1}^{n}{\left(q_{ij}-a_{ij}^{-}\right)}^{2}}$.

So, the total score is *C*_*i*_=*D*_*i*_^−^/*D*_*i*_^+^+*D*_*i*_^−^.

From the formulas above, 0 ≤ *C* ≤ 1. When *C*=1, the village has the optimized solution. When *C*=0, the village has the worst solution. When *C* is closer to 1, the village is closer to the optimized solution and has the highest score.

### 3.2. Data Sources and Evaluation System

#### 3.2.1. Data Sources

To ensure the consistency and reliability of the data, it is mainly from statistical yearbooks and statistics on government websites.

#### 3.2.2. Building of Element System

Based on the National Rural Revitalization Strategic Plan (2018–2022) and other relevant research, the element system tries to focus on key areas and links, such as rural economic efficiency, sustainable agricultural and rural development, narrowing the urban-rural gap, and leading a healthy and happy rural life. The five levels: thriving industries, social civility and etiquette, prosperity, effective governance, and pleasant living environments are regarded as first-level elements [29]. In terms of the selection of secondary elements [30] and according to the national rural revitalization strategy [31]implementation and effectiveness element system proposed by, and the results of the statistical analysis of the evaluation elements by the frequency analysis method by and, finally, following the principles of objectivity, continuity, availability, and consistency, a system of evaluation elements for the development level of high-quality rural development including 15 elements was constructed, as shown in Table 1.

Thriving industries are needed for high-quality rural development because whether the industry is prosperous or not will fundamentally determine the speed and efficiency of the rural development. Industrial prosperity has an overall impact on development. Industrial prosperity has a linkage effect and will have an overall impact on the high-quality development of rural areas. This paper will measure industrial prosperity from agricultural production efficiency, mechanization level, agricultural technology level, marketization of agricultural products, and agricultural structure.

General Secretary Xi Jinping has pointed out that a pleasant living environment with clear waters and green mountains are as good as mountains of gold and silver, and the maintenance and governance of the rural ecological environment is important for high-quality rural development and can effectively offer villagers a sense of security and improve the environment. The rural environment can guarantee a livable environment and rural production. Rural ecology is mainly to promote the establishment of a livable environment and ensure rural production, and ecological livability is mainly measured from the ecological environment and the living environment.

Social etiquette and civility can promote high-quality development in the countryside. Rural development cannot be separated from the promotion of social civility. It is rich in content because it involves historical culture and education. Rural style civilization is the software support for high-quality rural development, and high-quality rural development is inseparable from the construction of rural style civilization. Rural culture involves the development of rural public culture, cultural education construction, and the inheritance of excellent culture.

Effective rural governance is an important part of achieving high-quality rural development, and is also an important factor in promoting rural modernization, and to a certain extent, it can ensure the healthy and orderly development of rural society. Therefore, democratic practice, grassroots legal system, social morality, and balanced development are selected for measurement.

Prosperity is the fundamental goal and an important drive for high-quality development. In other words, it is important for development. The quality of life of rural residents is measured by their income, income gap with other areas, and living standards.

#### 3.2.3. Test of the Applicability of Element Analysis

Using SPSS25.0 to conduct KMO test and Bartlett test on the sample data, the results in Table 2 show that the KMO value is 0.696 (>0.5) and significant, so it is considered that there is a strong correlation between the original variables, that is, the sample data is suitable for factor analysis and Principal Component Analysis. Table 2 shows the Test of KMO and Bartlett.

#### 3.2.4. The Calculation of the Weight of Each Element

The data is analyzed for the cofactors and then variance contribution *p*_*k*_ and component score coefficient *β*_*i*_ as seen in Tables 3 and 4. Then use the formula to calculate the weight (see Table 1).

#### 3.2.5. Scores of the Traditional Villages

According to Table 5, the element of industrial prosperity has a larger weight, indicating that the representative villages have the most significant gap between them. An affluent life standard and effective governance are not far behind, and social civility and etiquette and a pleasant living environment rank last, indicating each village has a pleasant environment. As a result, the two elements have little effect on the weight of assessment indices. However, the factors related to farm economy, such as *A*_1_, *A*_2_, *A*_3_, have a greater impact on the comprehensive score, and other factors related to rural culture, such as *C*_1_, *C*_2_, *C*_3_, have the least impact on it.

Judging from the scores in Table 6, Pingzhai Village of Longchang Town comes in first place in terms of thriving industries, and Laidong Village finished last. Shihua Village and Laidong Village are ranked first in terms of pleasant living environments. Diliang Village and Ganxi Village come last. Ganxi Village is ranked first place in terms of social civility and etiquette, and Laidong Village is ranked last. Wengdang Village did the best in terms of effective governance, the worst is Shihua Village. Pingzhai Village of Longchang Town ranked first place in terms of prosperity, and Laidong Village is ranked last.

### 3.3. Optimized Approaches to Better Development

#### 3.3.1. Overview of Traditional Villages

**Figure 1:**
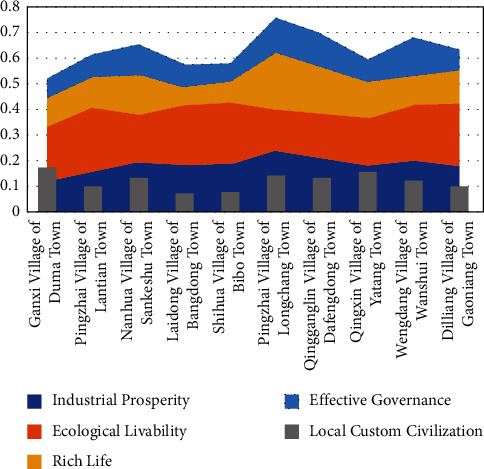
Cumulative scores for each village.

**Figure 2:**
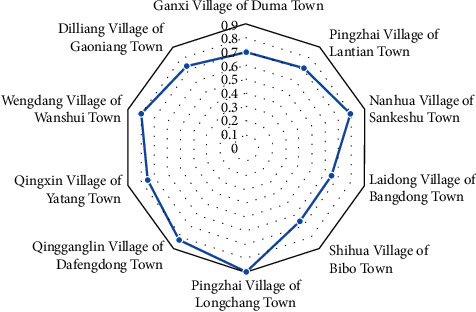
Ranking of each village.

Figure 1 shows the cumulative scores for each village, and Figure 2 shows the ranking of each village.

#### 3.3.2. Optimized Approaches to Industrial Prosperity

Figure 1 suggests that industrial prosperity occupies the highest proportion in all dimensions and levels. It is a very large influencing factor, which ranks first and is the most active in general. Villages with better industrial development have higher total scores. Pingzhai Village of Longchang Town, Qingganglin village, Wengdang Village, and Nanhua village are showing as having the highest scores, and analysis of these can therefore help with the development of Laidong village, Ganxi Village, Pingzhai Village of Lantian Town, and Diliang Village. In addition, the paper finds that efforts should be made to promote industrial prosperity from the following perspectives: First, win the tough battle to alleviate poverty. Second, consolidate the basis for agricultural development. Third, accomplish the key tasks of the primary phase of promoting high-quality development: thriving industries, pleasant living environments, prosperity, effective governance, social civility, and etiquette. Finally, allow top priority to an effective policy framework for agricultural and rural development priorities and mobilize resources to support the four priorities: personnel, resources, finance, and allocation of public services. Rural areas are the key to realizing the great rejuvenation of the Chinese nation in all respects, and farmers are the key to rural revitalization and eliminating poverty. In addition, industry must be allowed to develop by harnessing land resources to their full potential. With regards to the relationship between land-use transformation and rural transformation, referring to high-quality rural development as the foundation for a new type of rural reconstruction, the relationship can be deepened between land-use transformation and rural transformation development and reconstruction. The recessive form of land use and its changes should be the key to realizing high-quality rural development through controlling the transition of land use. Developing business is an important feature of high-quality rural development. The combination of land resources and rural industries is of great significance to the sustainable development of the rural economy and efficient use of resources. Therefore, it is urgent to control the hidden morphological changes of land use, enable innovative land management policies and regulations, and explore a new mode of land-use transformation combined with multifunctional agriculture for better rural development.

#### 3.3.3. Optimized Approaches to Pleasant Living Environments

Villages with more pleasant living environments get lower total scores, and most of them are ones with relatively backward industries and poor industrial development. It has something to do with their destructive economic development, or an implacable difference exists between their industry and environment. The results show that environment has a great impact on rural development. Generally, there is interaction between environment and growth. Factors such as complex geographical environment and land-use policy reveal the evolution of contemporary rural areas. Due to changing economic and social framework conditions, future demands on rural landscapes will also change [32]. As for Qiandongnan Miao and Dong Autonomous Prefecture, the backward economy, the sound environment, and the limited development of resources are responsible for their poor development. Population, rural infrastructure, and ecological environment are the main internal variables that determine the direction of rural evolution. Villages where people have a higher living standard have more sustainable development than that of less developed ones. Although villages located in the less developed areas economically have higher scores in terms of ecological environment, the negative impacts of population loss and economic recession on sustainable rural development are significant. Both large-scale socioeconomic and ecological environments have an impact on rural evolution. Therefore, ecologically, livability should not only refer to a pleasant living environment but should also refer to a spiritual well-being and, more importantly, good economic living standards.

#### 3.3.4. Optimized Approaches to Social Civility and Etiquette

Picture 1 shows that the overall coefficient score of rural civility and etiquette is not the highest, but it is always in a relatively stable position. The influence of rural civility and etiquette on villages is relatively stable. Therefore, it can have a direct or indirect effect on the high-quality development of rural areas so that the villages with lower scores in rural civility and etiquette get lower total scores. Therefore, it is necessary to explore the methods of developing rural civility and etiquette in villages. The development of rural civility and etiquette in China is greatly influenced by national and local policies. Also, it cannot be separated from the development of local culture. Therefore, rural civility and etiquette also need to be led by the government and subject to laws. It is difficult to maintain local culture and develop new culture if the former can no longer be protected and the latter is half formed. Limited living space and farmers' inability to express and develop cultural aspects are partly responsible for the issue. Culture is the foundation of rural development because decadent culture tends to bring about backward villages. Adequate living space is essential for the prosperity of rural culture, but these spaces need to be carefully planned to accommodate and develop local cultural needs. Despite China's rapid urbanization and industrialization, most Chinese people still live in the vast countryside areas or are still registered as rural residents. Rural civility and etiquette cannot be developed without economic growth, without keeping alive local culture, and without good leadership and the improvement of laws and regulations.

#### 3.3.5. Optimized Approaches to Effective Governance

Through the analysis of pictures 1 and 2, effective governance can guarantee high-quality rural development. Although the score of effective rural governance is relatively stable, the overall score of each village is relatively low and villages with better industrial development have higher scores regarding governance. But, overall scores of each village are not ideal, which greatly affects their development. So, it is necessary to explore in-depth the approach towards effective rural governance. Effective governance is an important part of advancing the modernization of the Chinese socialist governance system. The rule of law is the fundamental way to rural governance in China. To cultivate and apply the rule of law within rural jurisdictions must meet practical needs. Efforts should also be made to reform rural governance and promote the modernization of the rural governance system and capacity. The key to high-quality rural development is effective governance, which needs proper handling of issues such as ethical relationships within the governance process. The modernization of rural governance in the new era is the foundation for the strategy of high-quality rural development and the modernization of national governance. Self-governance, rule of law, rule of virtue, and rule of technology are the theoretical ideas behind functional, rural governance in the new era. The combination of autonomy, rule of law, rule of virtue, and the rule of technology is the only way forward for rural governance in the new era. Having an autonomous body, the rule of law, the rules of virtue, and the rational choice of technological transformation is the main contradiction of Chinese socialism within the new era of rural governance and the best combination of good rural governance.

#### 3.3.6. Optimized Approaches to Prosperity

Common prosperity will be an important factor as to whether villages can achieve high-quality development [33]. According to Figures 1 and 2, it is not difficult to find that the development level of common prosperity in each village is relatively low, but it is in the middle range in terms of dimensional scores. It is not conflicting because it is related to the small income gap within each interval. However, the existing level of common prosperity is difficult to support the level of its middle range, and it is necessary to promote common prosperity [34]. The high-quality development of the countryside is inseparable from the realization of common prosperity. Promoting market integration, improving the efficiency at a national level, and contributing support to the more backward areas. Allow the higher scoring villages, such as Pingzhai Village of Dafengdong Town, Qingganglin Village, Wengdang Village, and Nanhua Village, to help the relatively backward villages such as Laidong Village, Ganxi Village, Pingzhai Village of Lantian Town, and Diliang Village by implementing their successful model so that the strongest support weakest. In this way, the goal of common prosperity can be achieved. High-quality rural development is only possible with the process of urbanization. From the perspective of overall high-quality rural development achieving common prosperity, some villages with relatively good economic development conditions must attract more people [35]. For those villages with less advantageous economic conditions to achieve common prosperity, some of the small business economic activity provides local employment [36]. Also, they should allow to some extent for an outflow of population [37]. In this case, a moderate outflow of population will allow for more opportunities for the local people and gradually help them to achieve higher incomes.

## 4. Limitations and Consideration

Analysis of the status quo of 10 ethnic minority villages in Qiandongnan Miao and Dong Autonomous Prefecture, Guizhou Province reveals that there are limitations that seem so prevalent regarding rural revitalization.

1. Since 2019, the country has reformed land-use policies and implemented national land space planning, which has imposed stricter restrictions on land use. Meanwhile, the data collected in the third land survey carried out in the past two years is more accurate than that of ten years ago, so that the use of land for construction is relatively strict. The original land reserved for construction is also very limited, and it will be difficult to expand on this in future. Therefore, due to the control of the land at national level, many buildings of secondary and tertiary industries cannot be constructed, leading to difficulties in industrial development. Maybe in the future, urban-rural integration will be the way of rural revitalization.
2. According to our team's on-the-spot investigation of the villages, many villages are still at the planning stage, and the plan is difficult to carry out due to limited government funds. For example, Xijiang Qianhu Miao Village in Leishan County, Qiandongnan Prefecture, Guizhou Province, is rated as a 4-A level tourist attraction, which shows it is highly rated and is a very successful case of rural revitalization. It attracts millions of tourists every year who come to visit and experience the cultural customs of the local ethnic minorities. It also drives local economic development. For villages, tourism is usually needed to drive local development, but in fact, only 2–3 million yuan is invested in rural revitalization in each local village each year. Such a small amount of capital investment is like a drop in the ocean for a village with several hundred households.
3. Limited conditions allowing for development of the villages lead to difficulties in rural revitalization and construction. As Guizhou Province is located in the southwestern mountainous area of China, it naturally faces hardships and difficulties with regards to transportation. In particular, methods of transportation to and from many villages is small and inconvenient, making it difficult for the development of tertiary industry. With such limited condition, there are some difficulties occurring in rural spatial planning—procedural and distributive justice and value conflicts. In addition, the infrastructure of the village itself is also far from being perfect. According to our survey, although many villages are basically electricity covered, but the wires are overhead and not buried in the ground. Despite this, equipment allowing for running water is installed although the sewage treatment system is not perfect, and some waste water is discharged into natural water bodies directly. Although most roads have been developed and maintained, the roads are basically single lanes with limited parking. Fire safety aspects are not ideal and some living facilities are very old. Some villages have poor mobile phone signals and there are almost no facilities that could support the development of tertiary industry. According to the existing research, if a village wants to develop the local economy through tourism and other tertiary industries, the requirements for good basic local conditions, infrastructure, and supporting elements are demanding. For some traditional villages in Qiandongnan Miao and Dong Autonomous Prefecture, it takes considerable effort just to improve the living environment.

## 5. Conclusions

This paper forms a system for the evaluation of the rural development with 5 elements including social etiquette and civility, pleasant living environments, effective governance, prosperity, and thriving industries, and a total of 17 indicators. Based on statistics and data from the local government, a comprehensive evaluation of the rural revitalization in representative villages of Qiandongnan Miao and Dong Autonomous Prefecture is conducted through the factor analysis and TOPSIS method. The results show that the evaluation system and evaluation method can more scientifically measure the development of all the cities and villages. Therefore, the evaluation index system can generally be used. As a result, it can be promoted in other regions in order to provide feedback on the experiences for the rest of the country.

This paper uses this evaluation system to evaluate all data, and it can be found that: First, rural industrial development has an important impact on the implementation of the high-quality rural development strategy; second, the development in economically prosperous villages is significantly better than those with relatively underdeveloped economies, which are home to lower income and lower production output. Total scores for Laidong Village, Ganxi Village, Pingzhai Village of Lantian Town, and Diliang Village are relatively low. Third, there are large differences in resources regarding agricultural technology and power supplies for agricultural production between all villages, so joint efforts are required from the government and the local people. Fourth, democratic practice, the implementation of grass root policies, moral governance, and the inheritance of excellent and valued culture will each affect the overall scores of high-quality rural developments and affect the process. Therefore, these factors should not be underestimated. Finally, the ecological environment, living environment, and agricultural structure are generally not ideal in the villages of Qiandongnan Miao and Dong Autonomous Prefecture, they have multilayered characteristics, apart from that, local residents also play an important role in rural revitalization, especially leaders and key people [37], so universal and comprehensive management and joint efforts are needed in order to refine them [38–40].

## Figures and Tables

**Table 1:** Element systems for assessing the development of representative villages.

Goal	Primary element	Secondary element	Unit	Goal
Rural revitalization	Thriving industries *A* (0.346)	*A*_1_ efficiency of agriculture	%	0.098
		*A*_2_ mechanization of agriculture	—	0.083
		*A*_3_ industrialization of agriculture	%	0.064
		*A*_4_ marketization of agricultural products	%	0.056
		*A*_5_ structural agriculture	%	0.045
	Pleasant living environments *B* (0.145)	*B*_1_ ecological environment	—	0.069
		*B*_2_ living environment	%	0.076
	Social civility and etiquette *C* (0.084)	*C*_1_ development of public culture	—	0.018
		*C*_2_ culture and education	—	0.034
		*C*_3_ inheritance of outstanding cultures	—	0.032
	Effective governance *D* (0.169)	*D*_1_ form of democracy	%	0.017
		*D*_2_ rule of law at grassroots level	—	0.043
		*D*_3_ rule of virtue	—	0.054
		*D*_4_ coordinated development	—	0.055
	Prosperity *E* (0.256)	*E*_1_ income	RMB	0.098
		*E*_2_ income gap	RMB	0.083
		*E*_3_ living security	%	0.066

Note: Some elements derive from the data of the third China Agriculture Census.

**Table 2:** The test of KMO and Bartlett.

Amount of Kaiser–Meyer–Olkin	0.696

KMO and Bartlett's test	**123.854**

Approx. chi-square	**36**

Sig.	**0.**

**Table 3:** Contribution of variance.

Total variance										
	Elements	Initial eigenvalues			Extraction sums of squared loading			Rotation sums of squared loading		
		Totals	Variance percent	Accumulates %	Totals	Variance percent	Accumulates %	Totals	Variance percent	Accumulates %
Initial state	1	0.232	57.402	57.402	0.232	57.402	57.402	0.225	55.710	55.710
	2	0.060	14.903	72.305	0.060	14.903	72.305	0.019	4.586	60.296
	3	0.038	9.491	81.795	0.038	9.491	81.795	0.050	12.284	72.580
	4	0.033	8.232	90.027	0.033	8.232	90.027	0.014	3.340	75.920
	5	0.015	3.800	93.827	0.015	3.800	93.827	0.035	8.534	84.454
	6	0.010	2.472	96.299	0.010	2.472	96.299	0.023	5.685	90.138
	7	0.009	2.266	98.565	0.009	2.266	98.565	0.034	8.427	98.565
	8	0.005	1.133	99.699						
	9	0.001	0.301	100.000						
	10	7.284E-17	1.801E-14	100.000						
	…	…	…	…						
	47	−5.347E-17	−1.322E-14	100.000						

**Table 4:** Rotating ingredients and score coefficient.

Elements	Element 1 (score coefficient)	Element 2 (score coefficient)	Element 3 (score coefficient)	Element 4 (score coefficient)	Element 5 (score coefficient)	Element 6 (score coefficient)	Element 7 (score coefficient)
*A*_1_					0.755 (0.116)		
*A*_2_							0.519 (0.117)
*A*_3_		0.848 (0.493)					
*A*_4_		−0.771 (0.146)					
*A*_5_					−0.679 (0.034)		
*B*_1_	0.984 (0.08)						
*B*_2_						−0.487 (0.055)	
*C*_1_					0.637 (0.025)		
*C*_2_			−0.48 (−0.059)				
*C*_3_	0.978 (−0.027)						
*D*_1_		0.68 (0.101)					
*D*_2_				0.434 (0.019)			
*D*_3_				0.568 (0.05)			
*D*_4_			0.465 (0.023)				
*E*_1_						0.538 (0.18)	
*E*_2_					0.627 (0.261)		
*E*_3_							0.537 (0.012)

**Table 5:** Total scores of the representative villages and ratings.

Villages	Total scores	Ratings
	Similarity measure	
Ganxi Village of Duma Town 2817	0.694	8
Pingzhai Village of Lantian Town 3205	0.712	7
Nanhua Village of Sankeshu Town 5200	0.791	4
Laidong Village of Bangdong Town 1722.5	0.649	10
Shihua Village of Bibo Town 2458.31	0.656	9
Pingzhai Village of Longchang Town 5290	0.898	1
Qinganglin Village of Dafengdong Town 5248	0.825	2
Qingxin Village of Yatang Subdistrict 4560	0.752	5
Wengdang Village of Wanshui Town 5220	0.803	3
Diliang Village of Gaoniang Town 4125	0.733	6

**Table 6:** Scores of representative villages in every aspect.

Villages	Totalscores	Thrivingindustries	Pleasant livingenvironments	Social civility andetiquette	Effectivegovernance	Prosperity
Ganxi Village of Duma Town	0.693	0.127	0.206	0.175	0.073	0.112
Pingzhai Village of Lantian Town	0.712	0.156	0.249	0.099	0.087	0.121
Nanhua Village of Sankeshu Town	0.791	0.194	0.182	0.137	0.121	0.157
Laidong Village of Bangdong Town	0.649	0.182	0.233	0.075	0.088	0.071
Shihua Village of Bibo Town	0.656	0.187	0.238	0.077	0.072	0.082
Pingzhai Village of Longchang Town	0.898	0.237	0.161	0.142	0.139	0.219
Qinganglin Village of Dafengdong Town	0.825	0.209	0.172	0.133	0.128	0.183
Qingxin Village of Yatang Subdistrict	0.752	0.181	0.183	0.158	0.088	0.142
Wengdang Village of Wanshui Town	0.803	0.201	0.213	0.122	0.159	0.108
Diliang Village of Gaoniang Town	0.733	0.177	0.242	0.098	0.084	0.132

## Data Availability

The dataset can be accessed upon request.
